# Supplementary material for: Prevalence of and related risk factors in oral mucosa diseases among residents in the Baoshan District of Shanghai, China
Source: PeerJ. 2020 Feb 24;8:e8644. doi: 10.7717/peerj.8644 (PMC7045885; doi:10.7717/peerj.8644)
Supplement: Supplemental Information 2 [file peerj-08-8644-s002.doc]

Epidemiological investigation and analysis of oral mucous membrane disease in Shanghai residents

Family member list

District Street Residents' committees

| Family  number | Questionnaire number | Name | The relationship with the head of the household |
| --- | --- | --- | --- |
|  |  |  |  |
|  |  |  |
|  |  |  |
|  |  |  |
|  |  |  |
|  |  |  |

Oral mucosa health questionnaire

Name Sex Age Date of birth

Number census register

Oral mucosa 【Clinical diagnosis】

【Oral symptoms】 【lesion location】 0 normal mucosa

1. no abnormality 1 lip red rim 1 chronic lip and keratitis

1 ulcer 2 mouth spout 2 map tongue

2 mottled 3 lips mucous membrane 3 groove tongue

3 congestion 4 buccal mucosa 4 atrophic glossitis

4 erosion 5 back of tongue 5 herpes simplex

5 false membrane 6 tongue and abdomen 6 allergic stomatitis

6 scab 7 mouth bottom 7 behcet's disease

7 atrophy 8 hard / soft palate 8 aphthous ulcer

8 chaps 9 alveolar bones/gums 9 traumatic ulcers

9 scales 10 lichen planus

10 Blisters、bullae and pustules 11 white spots

11 Pimples and papules 12 pemphigus

12 Nodule、tumor 13 discoid lupus erythematosus

13 Necrotic gangrene 14 burning mouth syndrome

14 periodontal damage 15 oral submucosal fibrosis

16 tumors

17 others

【Clinical examination】


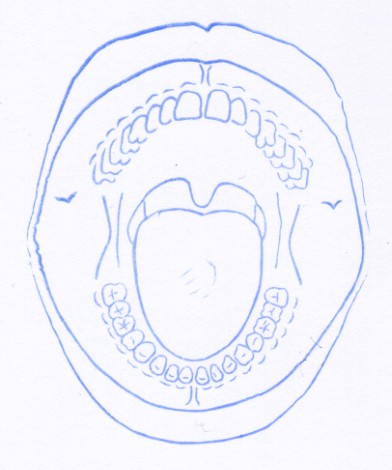

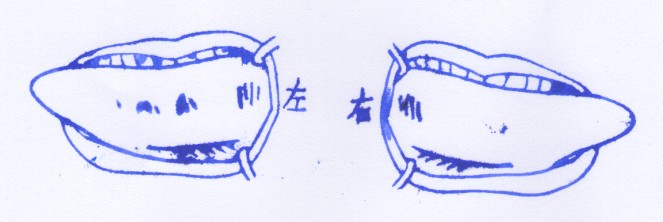


Comprehensive situation questionnaire (family members over 15 years old)

Name Number

**The first part: demography**

A1 nationality, 1 han nationality, 2 others, please specify

A2 domicile of origin:

A3 years of residence in Shanghai: years

A4 level of education

1 illiterate 2 primary school 3 junior middle school 4 senior high school/technical secondary school 5 major/junior college 6 graduate students or above

A5 marital status

1 unmarried 2 married 3 remarried 4 divorced 5 widowed

A6 career

1 worker 2 farmer 3 soldier 4 civil servant 5 scientific and technical personnel 6 medical personnel 7 teachers 8 finance 9 business service personnel 10 housewives 11 retirees 12 unemployed 13 students

**The second part: smoking**

B1 are you currently smoking cigarettes (including cigarettes, hand cigarettes, pipes, shisha, chewing tobacco, snuff, cigars, etc.)?

1 yes. 2 used to smoke, but now he has given up smoking. 3 no

B2 when did you start smoking?  one full year of life

B3 like smoking. How long have you been smoking?

B4 average number of smoking days per week?  day

How much does B5 usually smoke a day?  branch

**The third part: alcohol consumption**

C1 drinking?

1 yes 2 no. 3 has given up drinking

C2 if drinking, how long has it been since the beginning of drinking?

Types and amounts of alcohol consumed

|  | Types | a Whether or not to drink:  1 yes, 2 no | b Drinking frequency (frequency/week) | c Average amount per drink |
| --- | --- | --- | --- | --- |
| C3 | liquor (≥42 degrees) |  |  |  |
| C4 | liquor (< 42 degrees) |  |  |  |
| C5 | beer |  |  |  |
| C6 | Yellow rice wine |  |  |  |
| C7 | wine |  |  |  |

**The forth part: diet**

D1 eating habits (multiple choices)

1 light 2 salted products 3 spicy 4 hot 5 others

D2 fruit consumption frequency

1 eat every day 2 occasionally 3 rarely or not

D3 frequency of vegetable consumption

1 eat every day 2 occasionally 3 rarely or not

**The fifth part: health**

E1 Has been diagnosed with hypertension by doctors in community or above hospitals?

1 yes 2 no

E2 Have you ever been diagnosed with diabetes by a doctor in a community or above hospital? (investigators note: gestational diabetes was not included.)

1 yes 2 no

E3 Has any of the following chronic diseases been diagnosed by doctors at community or above hospitals? (multiple choices)

1 no chronic disease has been diagnosed

2 coronary heart disease (CHD)

3 Cerebrovascular diseases (such as cerebral hemorrhage, subretinal hemorrhage, cerebral thrombosis, cerebral embolism, etc.)

4 Immune system diseases

5 tumor

6 for the other, please care for the care

E4 Have any of your grandparents, parents or siblings been diagnosed with the following chronic diseases by doctors in the community or above? (multiple choices)

1 no chronic disease has been diagnosed

2 high blood pressure

3 coronary heart disease (CHD)

4 Cerebrovascular diseases (such as cerebral hemorrhage, subretinal hemorrhage, cerebral thrombosis, cerebral embolism, etc.)

5 diabetes

Immune system diseases

7 tumor

8 Care for the other, please care for the care_________

**The sixth part:** mental state

The Following questions are about your feelings or experiences in the past 12 months. Please answer as honestly as possible.

F1 Do you feel lonely, nervous, worried or scared?

1 never 2 rarely 3 sometimes 4 often 5 always

F2 Do you feel a lot of pressure from work, study or life?

1 never 2 rarely 3 sometimes 4 often 5 always

F3 What is your relationship with your family (e.g. parents, spouse, children)?

1 very good 2 good 3 general 4 bad 5 too bad

6 living alone without family

F4 Do you have very close friends?

1 has no 2 1-2 3 3-5 4 5 or more

F5 How often do you usually visit your good friends or family?

1 every day 2 1 week 3 2-4 weeks 4 4weeks or more

F6 In the past 12 months, have you encountered anything that hit you hard?

1 no 2 no t. 3 refused to answer
